# Quantitative Prediction and Analysis of Rattle Index Using DNN on Sound Quality of Synthetic Sources with Gaussian Noise

**DOI:** 10.3390/s24165128

**Published:** 2024-08-08

**Authors:** Jaehyeon Nam, Seokbeom Kim, Dongshin Ko

**Affiliations:** AI & Mechanical System Center, Institute for Advanced Engineering, Youngin-si 17180, Republic of Korea; jaehyeon@iae.re.kr (J.N.); sbkim@iae.re.kr (S.K.)

**Keywords:** BSR (Buzz, Squeak, Rattle), ANOVA, hold-out, K-fold cross validation, deep neural network

## Abstract

This study researched the prediction of the BSR noise evaluation quantitative index, Loudness N10, for sound sources with noise using statistics and machine learning. A total of 1170 data points was obtained from 130 automotive seats measured at 9-point positions, with Gaussian noise integrated to construct synthetic sound data. Ten physical quantities related to sound quality and sound pressure were used and defined as dB and fluctuation strength, considering statistical characteristics and Loudness N10. BSR quantitative index prediction was performed using regression analysis with K-fold cross-validation, DNN in hold-out, and DNN in K-fold cross-validation. The DNN in the K-fold cross-validation model demonstrated relatively superior prediction accuracy, especially when the data quantity was relatively small. The results demonstrate that applying machine learning to BSR prediction allows for the prediction of quantitative indicators without complex formulas and that specific physical quantities can be easily estimated even with noise.

## 1. Introduction

BSR (Buzz, Squeak, Rattle) noise is a common quality issue found in interior parts for automobiles, with over 50% of these issues occurring in panels, seats, and doors of automobiles [1,2,3]. Addressing consumer complaints resulting from BSR noise requires substantial costs for improvements. In the structural domain, BSR noise is linked to performance degradation and durability issues in components. BSR noise is classified into Buzz, Squeak, and Rattle problems. Buzz and squeak problems have a clearly structural mechanism, allowing for established theoretical and interpretive approaches to improvement methods. Particularly, studies on friction-induced noise focus on dynamic instability mechanisms that occur in systems based on linear theory, leading to mechanism-based solutions.

Kang et al. [4] developed a comprehensive mathematical mechanism for dynamic instability in brakes, providing a theoretical understanding of friction-induced noise. Nam et al. [5] analyzed the mechanism of the point contact friction model for friction-induced noise using a pin-on-disk friction system, which efficiently described the cause of friction-induced noise by describing the characteristics of the friction curve through experiments. Despite extensive research on friction-induced noise based on linearization theory through various applications, analyzing the mechanism of rattle noise remains challenging because of its nonlinear characteristics. 

Rattle can be described mathematically through an impact oscillator that includes Hertz’s contact model. However, researching rattle is challenging owing to extreme nonlinearities such as chaos [6,7]. Shin et al. [8] introduced a dynamic stiffness analysis technique, a degradation BSR analysis technique, and a direct virtual method developed from the BSR perspective to ensure the robustness of the BIW (Body-in-White) body system—a load transfer medium—and the corresponding modules for each part. Lee et al. [9] improved the E-Line method, commonly used to predict BSR noise, by utilizing a statistical method to determine the tolerances between parts expressed as dispersion and dynamic deformations. To directly express the behavior of rattle noise in the seats of autonomous vehicles, Kim et al. [10] calibrated an analysis model based on sinusoidal wave experiments and described the location and characteristics of impact noise through explicit analysis. Despite advancements in hardware and software enabling quicker analytical approaches to simulation-based studies on the rattle, significant time and cost are still required for these analyses. BSR mainly occurs in automotive interior parts and is evaluated during the final phase of performance verification of automotive seats to evaluate quality. Choi et al. [11] analyzed BSR characteristics after performing the excitation and operating durability tests on automotive seats and tracked the major noise sources. In another study, they analyzed the BSR vibration characteristics of the seat cushion frame before and after durability tests to assess how changes in the stiffness of the frame affected the BSR characteristics [12]. Wan et al. [13] conducted a study on an efficient noise diagnostic method using the STRE-VK method, which calculated measurement criteria for identifying various types of BSR by separating signal components and demonstrated that BSR could be predicted based on signal processing. 

Predicting BSR is challenging and does not provide a clear solution. Furthermore, comprehensive system analysis requires significant cost and time. Solutions are mainly carried out using experimental measurement methods, which require complex systems, expensive equipment, and engineers with expertise because the relevant regulations and calculation methods are complex.

Deep learning has surpassed human cognitive abilities in various fields through its rapid advancements. Algorithms built on nonlinearity achieve highly accurate predictions for unstructured data. Predictions using deep learning do not require equations of motion and rely solely on data, encompassing uncertainty and nonlinearity without the need for complex calculations. Wiercioch et al. [14] proposed a novel deep neural network (DNN)-based model to predict the characteristics of molecules and demonstrated accurate prediction for chemical characteristics. Additionally, Yu et al. [15] proposed a strategy to compromise the correlation between output variables through shared and separated parts by suggesting an MD-DNN (Multi-channel Decoupled DNN) model. DNNs are widely used in various fields to predict nonlinear systems.

In vibration analysis, deep learning is used to predict and analyze vibrations in numerous applications. Nam et al. [16] visualized the chaos phenomenon—the most complex phenomenon in dynamical systems—using various signal processing methods. They also described how recurrence plots can be used to classify chaos phenomena utilizing convolutional neural networks (CNNs). Recurrence plots require a reconstructed phase space to address self-crossing issues, but it is challenging to reconstruct the ideal geometric dimensions of complex trajectories with noise, such as real-world phenomena [17]. Thus, predictions and classifications based on experimental data are expected to provide the most straightforward and purposeful direction for BSR studies. Huang et al. [18] proposed a theoretical architecture for diagnosing acoustic faults based on time-frequency analysis and machine learning using Support Vector Machine (SVM) techniques. They presented research results on fault identification based on signals measured using smartphones and discussed the accuracy of their results. 

BSR evaluation is determined based on Loudness N10, a quantitative metric. Since the calculation method for BSR evaluation is complex and requires expensive equipment and specialized software, predicting and verifying BSR characteristics in the design and development phases of components proves challenging. Conversely, it is impossible to obtain substantial BSR data from similar systems except from relevant development companies. Furthermore, even for these companies, it is unlikely to acquire substantial data through measurement methods. The sound source in the field inherently includes variability, which can differ from the ideally measured noise. Therefore, making predictions based on machine learning faces the following challenges: insufficient data, issues with informal data such as noise, and data classification problems. 

**Figure 1:**
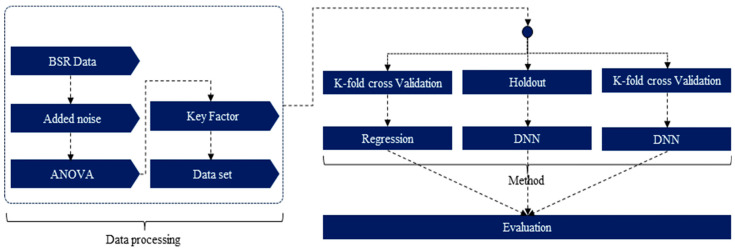
Flow diagram of prediction method for Loudness N10.

In this study, we simulated real-world noise to reconstruct seat noise to predict Loudness N10 a quantitative metric used to evaluate BSR noise. Particularly, we aimed to estimate the BSR characteristics of the developed system by predicting Loudness N10 a quantitative metric for BSR in an anechoic chamber through simple field tests using noise containing sound sources. Loudness N10 predictions are based on statistics, and in this study, we described a method for predicting quantitative metrics solely based on the characteristics of physical quantities without requiring special equipment or calculations. We analyzed significant physical quantities from a statistical perspective and the characteristics of Loudness N10 through correlation analysis and derived two significant physical quantities. Data augmentation was not utilized as it can distort data, and the method of increasing the amount of data was not used because it is a common method for enhancing the performance of models. Instead, we employed the K-fold cross-validation technique to address data limitations. Loudness N10 predictions were made using the physical quantities analyzed through a DNN. Figure 1 illustrates the flow diagram of the prediction procedure and performance verification of the proposed method.

## 2. Method

### 2.1. Construction of BSR Dataset and Physical Quantity Information

**Figure 2:**
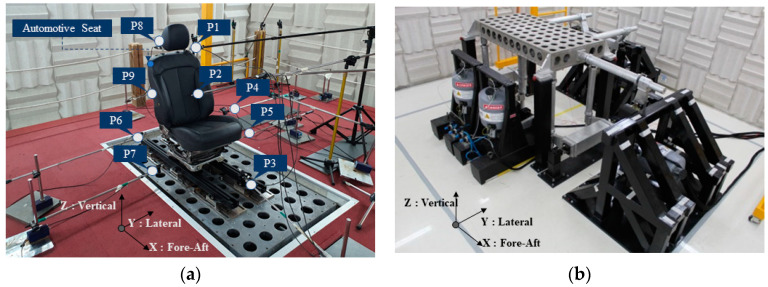
Experimental setup: (**a**) sensor position; (**b**) test equipment.

BSR measurements and Loudness N10 calculations were performed based on GMW 14011, as illustrated in Figure 2 [19]. The BSR data were extracted from positions 150 mm away from each point on the car seat, as shown in Figure 2a, in accordance with GMW 14011. A multi-axis silent shaker was used, as depicted in Figure 2b. The background noise of the anechoic chamber was within 30 dB(A) under the operating conditions of the shaker, and the environmental chamber allowed for temperature control from −40 °C to 50 °C. The experimental conditions of the configured dataset were measured at low temperature (−20 ± 5 °C), room temperature (23 ± 5 °C), and high temperature (50 ± 5 °C). 

Loudness N10 was calculated using software(ArtemiS Classic V12) based on Zwicker Loudness. BSR data were measured using nine microphones across the 130 different seat models used, resulting in a total of 1170 data points. The data used varied in environmental conditions, such as temperature and seat position, during the measurement process. Since this study aims to estimate Loudness N10, which requires complex calculations based on various physical quantities related to sound quality and acoustics, environmental conditions were not considered. However, the same test method was used for all measurement conditions. An exciter with operating background noise less than or equal to 30 dB(A) and a 300 Hz high-pass filter were utilized in the experiment. Loudness N10 estimation was performed by analyzing the characteristics of a total of ten physical quantities related to sound quality and acoustics. Each physical quantity was based on the lowest level (N10) in the top 10% positions. Ten physical quantities relating to sound quality and acoustics were used: Loudness (M1), 3rd octave (M2), sound pressure level (M3), fluctuation strength (M4), Roughness (M5), Sharpness (M6), Tonality (M7), Harmonic distortion (M8), Speech intelligibility index (M9), and Articulation index (M10). Given the significance of magnitude in BSR, the selection of the physical quantities was defined as the physical quantities for sound pressure level and those that determine emotional quality. 

The measured signals contain noise due to the external environment and structural issues. Data measurement involves considering the measurement process and analyzing the signals through a filtering process using specialized hardware and software for system characteristics. Noise can be implemented using various methods, but in numerical analysis methods, it is generally implemented using Gaussian noise. The probability density function of the noise applied to BSR sound sources is defined as follows:

(1) $$f(z)=1/\sigma \sqrt{2\pi }exp(-(z-\mu {)}^{2}/2\sigma^{2}).$$

**Figure 3:**
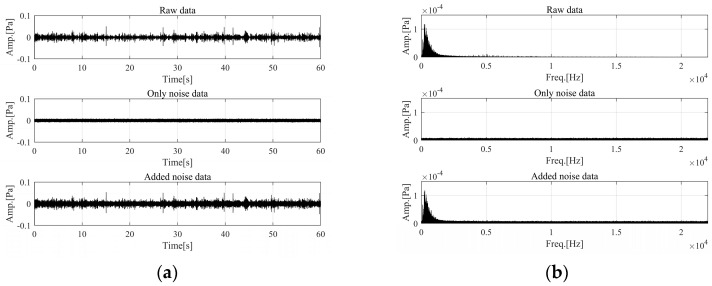
Characteristics of added noise in raw data: (**a**) time domain; (**b**) frequency domain.

In this equation, σ and μ represent the standard deviation and mean of the noise signal, respectively, and z denotes the noise signal. Noise was implemented using a Gaussian random distribution, and the standard deviation was modeled at the 2/3 level of the basic data. The characteristics of the signals with noise are illustrated in Figure 3. Figure 3a,b illustrate the results in the time domain and frequency domain, respectively. Gaussian noise was introduced into the raw data to exhibit characteristics of the added noise that did not exist previously. Particularly in the frequency domain, the characteristics of the added noise are exhibited across all frequencies except the fundamental frequency. 

Each physical quantity was normalized according to the physical quantity calculation method because the absolute magnitude varies depending on the calculation method. Common normalization methods include the min–max normalization method and the z-score normalization method. The z-score method is suitable for handling outlier problems and is sensitive to the mean and standard deviation of the data. However, the constructed data were measured at equal intervals using microphones with similar specifications at nine positions. Since the microphones had similar characteristics, the possibility of outliers occurring is minimal. Thus, the min–max normalization was performed. The normalization results are not a conclusion of this study, and the relationship between Loudness (M1) and each metric was intuitively compared using the minimum value (0) and maximum value (1). Although 130 data points were analyzed through the data analysis, only the results for representative samples were described. Table 1 illustrates the results of the samples containing normalized noise. 

As shown in the normalized results, Loudness exhibits extremely similar characteristics to the acoustic physical quantities M2, M3, M4, M5, and M6. Conversely, Loudness shows contrasting results with M7, M9, and M10. Alternatively, Loudness is presumably determined by the magnitude of the noise and the frequency of the sound. Although complex factors enable a precise analysis of systems, they complicate the polynomials. Hence, it is necessary to exclude physical quantities with low impact. Therefore, covariance analysis and correlation analysis were performed to define the relationship for each physical quantity and derive significant factors. 

### 2.2. Variables for the Physical Quantity Correlation Analysis and Determination of the Variables

Physical quantities calculated using different methods represent the characteristics of sound quality. Thus, the selection of physical quantities analyzes the correlation of related variables to derive the final physical quantities that will be used in regression and deep learning. Correlation analysis examines the strength of the linearity between the physical quantities and identifies the presence of linear relationships as a statistical result. It also defines the correlation between variables by performing covariance analysis and defines the relationship based on the levels of variables, regardless of units. A positive correlation exists between two variables when an increase in the value of one variable corresponds to an increase in the value of the other. Conversely, a negative correlation occurs when an increase in one variable results in a decrease in the value of the other. A covariance of zero indicates that the two variables are independent of each other. The results of the covariance analysis are listed in Table 2. 

Since the results of the covariance analysis define the relationship based on the level of the variables, they were expressed based on the normalized physical quantities. As shown in the covariance analysis results, each physical quantity exhibits a correlation with each other. Similar to the normalized data analysis results, the covariance analysis results indicate a correlation of approximately 0.07 between the magnitude-based quantities M2 and M3 and the physical quantities corresponding to frequency characteristics M4, M5, and M6. Considering vocal aspects, BSR noise is an unclear signal, suggesting that voice-related metrics may exhibit a high negative correlation. Since covariance does not include the degree of the relationship (the degree of the relationship according to the level of two variables), the relationship between the two variables was analyzed through correlation analysis. However, the previous covariance analysis was performed based on normalization to minimize the error in the deviation of levels. Therefore, it can be predicted that the correlation analysis results will exhibit similar characteristics to the normalized covariance analysis results. The results are listed in Table 3.

The analysis of correlation coefficients was performed using multiple correlation analyses for a total of ten physical quantities. The sample correlation coefficient indicates the linear correlation between variables. The results of the correlation analysis for each physical quantity demonstrated that the physical quantities related to the sound pressure level (M2, M3) exhibit the highest linear correlation, and the physical quantities corresponding to frequency characteristics (M4, M5, M6) also exhibit high linear correlation. Roughness and Sharpness demonstrated relatively high correlations, likely attributed to the low-frequency and high-frequency characteristics due to Gaussian noise instead of the correlation of the pure system. Figure 3 illustrates the correlation analysis results for Loudness N10 of the data with and without noise. 

**Figure 4:**
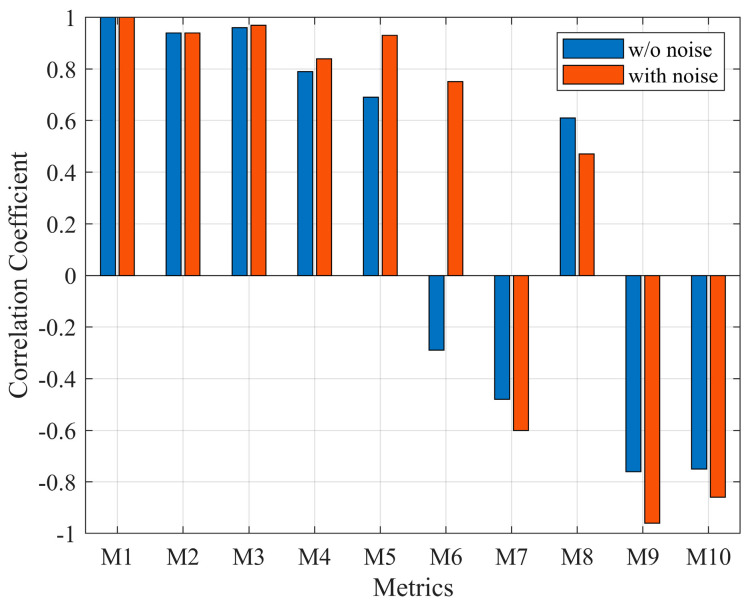
Comparison of correlation coefficient.

As illustrated in Figure 4, metrics related to the sound pressure level equally exhibited high correlations regardless of the presence or absence of noise. However, Roughness (M5) and Sharpness (M6), which correspond to the frequency characteristics, showed relatively low correlations in the absence of noise, while they exhibited high correlations when noise was present. This outcome can be attributed to the characteristics of Gaussian noise, which adds noise across the entire region. Hence, the correlation between Sharpness, which represents high-frequency characteristics, and Roughness, which represents low-frequency regions, increased. Therefore, sound pressure level (M2), which exhibits a high correlation with Loudness N10 regardless of the presence of noise, and fluctuation strength (M4), which can partially reflect the frequency characteristics, were selected as effective factors. 

### 2.3. Method of K-fold Cross-Validation

**Figure 5:**
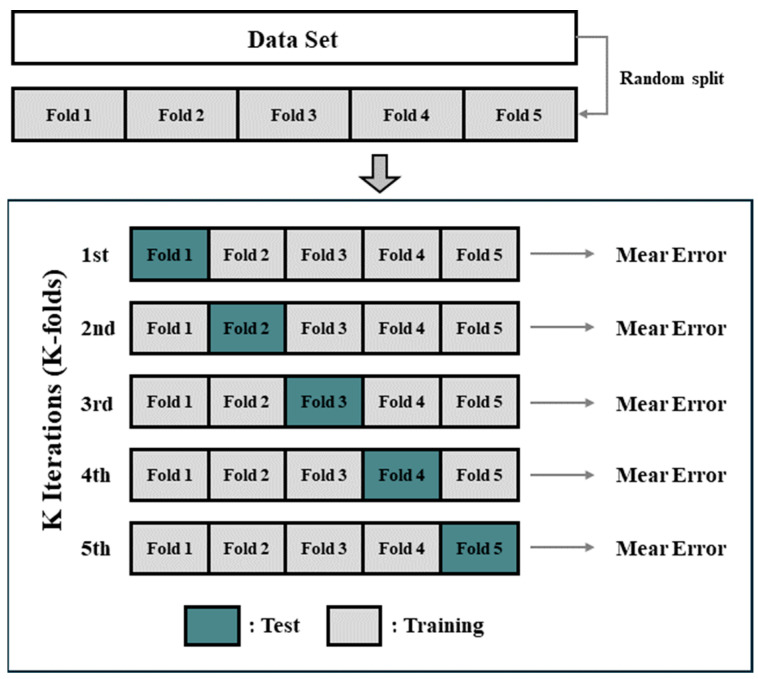
Schematic of K-fold cross-validation.

K-fold cross-validation is a method that evaluates a model by randomly partitioning the dataset into k sub-groups. It uses one of the sub-groups as the test data and the remaining k-1 sub-groups as the training data. This was repeated k times. The model is evaluated based on the average prediction error derived from each iteration. Typically, five or ten is used as the value of k to balance (Trade-off) the bias and variance of the regression model [20]. In general regression models, overfitting may occur, which only reflects biased characteristics. The K-fold cross-validation method can prevent this issue by randomly partitioning the dataset into training and test data and building and evaluating the model k times. Since BSR signals are collected during the final stage of the process, it is impractical to obtain a large amount of data. K-fold cross-validation is a representative method that leverages all data for both training and testing, thereby enabling the creation of a more generalized model and effective detection of overfitting and underfitting. Consequently, to address the issue of limited data, we employed K-fold cross-validation in this study, as illustrated in Figure 5.

Regression models can be divided into linear and nonlinear models, depending on the distribution of the data. No particular model is superior to the others. Rather, it is important to select the optimal model based on the type of data. In this study, the final model was selected by comparing the multiple linear regression model and a multiple nonlinear regression model. 

Multiple linear regression is a regression analysis technique that models the linear relationships between a dependent variable and two or more independent variables. The multiple linear regression model is expressed using a linear equation, as shown in the equation below. Y and $x_{i}$ are both independent variables. ${Β}_{i}$ is a regression coefficient and represents the influence of each independent variable.

(2) $$y=\beta_{0}+\beta_{1}x_{1}+\beta_{2}x_{2}+\cdots +\beta_{n}X_{n}$$

Linear regression uses the method of least squares, which minimizes the sum of the squares of the residuals, to estimate the regression coefficient. However, as the number of independent variables increases, multi-collinearity may occur due to the correlations between the variables. Hence, the variance of the least squares regression coefficient estimates increases, thus reducing the stability of the prediction accuracy of the regression equation [21].

In this study, a nonlinear regression model in the form of an exponential function was constructed through logarithmic transformation, as shown in the equation below. y and $x_{i}$ are both independent variables. ${Β}_{i}$ is a regression coefficient and represents the influence of each independent variable.

(3) $$y=\beta_{0}{x_{1}}^{\beta_{1}}{x_{2}}^{\beta_{2}}\cdots {x_{n}}^{\beta_{n}}$$

When there is a nonlinear relationship between an independent variable and a dependent variable, logarithmic transformation can be used to model this relationship linearly, making it a linear relationship. The regression coefficient of the linear model can be derived by applying the least squares method. Logarithmic transformation can linearly transform variables using natural logarithms, as shown in Equation (4).

(4) $$Lny=ln\beta_{0}+\beta_{1}{lnx}_{1}+\beta_{2}{lnx}_{2}+\cdots +\beta_{n}{lnx}_{n}$$

Here, the regression model can be expressed as Equation (5) for i datasets through matrix transformation.

(5) $$\left[\begin{array}{c} {Lny}_{1}\\ {lny}_{2}\\ \vdots \\ {lny}_{3} \end{array}\right]=\left[\begin{array}{ccccc} 1 & {lnx}_{1_{1}} & {lnx}_{2_{1}} & \cdots & {lnx}_{n_{1}}\\ 1 & {lnx}_{1_{2}} & {lnx}_{2_{2}} & \cdots & {lnx}_{n_{2}}\\ \vdots & \vdots & \vdots & \ddots & \vdots \\ 1 & {lnx}_{1_{i}} & {lnx}_{2_{i}} & \cdots & {lnx}_{n_{j}} \end{array}\right]\left[\begin{array}{c} ln\beta_{0}\\ \beta_{1}\\ \beta_{2}\\ \vdots \\ \beta_{n} \end{array}\right]$$

Assuming $Y=X\underline{\beta }$, the least squares estimate can be expressed as shown in Equation (6) when $(X^{\prime }X{)}^{-1}$ exists [22]. The regression coefficient is determined through Equation (6). If Equation (6) is substituted into Equation (4), reverse exponential transformation can be performed to derive a multiple nonlinear regression equation similar to Equation (3).

(6) $$\underline{Β}=\left[\begin{array}{c} ln\beta_{0}\\ \beta_{1}\\ \beta_{2}\\ \vdots \\ \beta_{n} \end{array}\right]=(X^{\prime }X{)}^{-1}X^{\prime }Y$$

### 2.4. Machine Learning Model 

**Figure 6:**
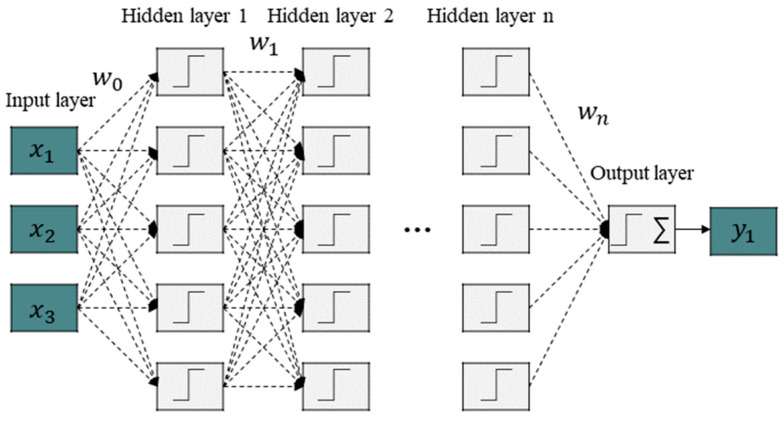
Structure of traditional DNN model.

Since deep learning is performed based on data, a substantial amount of data is generally required to improve accuracy. Accuracy typically improves with the increase in network depth, and an optimized model can be constructed through careful tuning of hyperparameters. A DNN, also known as a feedforward neural network or a multi-layer perceptron, is a neural network that has two or more hidden layers [23,24,25]. As illustrated in Figure 6, the DNN described in the example has three input dimensions and five neurons in the hidden layers. The output of the hidden layers is expressed as follows:

(7) $${z_{i}}^{1}=\sigma ({w_{i,1}}^{0}\;x_{1}+{w_{i,2}}^{0}\;x_{2}+{w_{i,3}}^{0}\;x_{3}+{w_{i,4}}^{0}\;x_{4}+{b_{i}}^{0})\;,\;(i=1,2,\ldots ,5)$$

In this equation, ${z_{i}}^{1}$ is the output of the i-th neuron of hidden layer 1. δ denotes the activation function, and ReLU is typically used as the activation function [26,27]. ${W_{i,j}}^{0}$ is the connection weight between the j-th input and the i-th neuron of hidden layer 1. Additionally, ${b_{i}}^{0}$ denotes the bias of the i-th neuron in hidden layer 1. Assuming there are n neurons in layer k−1, the output of the i-th neuron in layer k is calculated as follows:

(8) $${z_{i}}^{k}=\sigma (\sum_{j=1}^{n}{w_{i,j}}^{k-1}\;{z_{1}}^{k-1}+{b_{i}}^{k-1})\;,\;(k=1,2,\ldots ,K)$$

The feedforward neural network performs computations using the outputs of preceding layers, beginning with the input layer and going through to the results of the output layer. The neural network uses a loss function to measure the deviation between the predictions made by the model and the actual values and utilizes the gradient descent method to update the weights and biases of each layer to gradually bring the prediction values of the model closer to the actual values. The regression loss function for the K-th layer is calculated as follows:

(9) $$L(w,b,x,y)=\frac{1}{2}∥z^{K}-y{{∥}_{2}}^{2}=\frac{1}{2}∥\sigma (w^{K-1}z^{K-1}+b^{K-1})-y{{║}_{2}}^{2}$$

In this equation, $z^{K}$ represents the predicted value of the DNN model, and y denotes the actual value. The gradient of the loss function can be calculated as follows:

(10) $$\frac{\partial L(w,b,x,y)}{\partial w^{K-1}}=\frac{\partial L(w,b,x,y)}{\partial z^{K}}\frac{\partial z^{K}}{\partial w^{K-1}}=(z^{K}-y)⊙\sigma \prime (w^{K-1}z^{K-1}+b^{K-1})(z^{K-1}{)}^{T}$$

(11) $$\frac{\partial L(w,b,x,y)}{\partial b^{K-1}}=\frac{\partial L(w,b,x,y)}{\partial z^{K}}\frac{\partial z^{K}}{\partial b^{K-1}}=(z^{K}-y)⊙\sigma \prime (w^{K-1}z^{K-1}+b^{K-1})$$

In this equation, ⊙ denotes the Hadamard product. 

Assuming $\delta^{k}=\partial L(w,b,x,y)/\partial z^{k}=(z^{K}-y)⊙\sigma \prime (w^{K-1}z^{K-1}+b^{K-1})$ as the inactive output, the inactive output of the K-th layer, $z^{K}$, can be expanded as follows:

(12) $$\delta^{k}=\frac{\partial L(w,b,x,y)}{\partial w^{k}}=\frac{\partial L(w,b,x,y)}{\partial z^{K}}\frac{\partial z^{K}}{\partial z^{K-1}}\frac{\partial z^{K-1}}{\partial z^{K-2}}\ldots \frac{\partial z^{K+1}}{\partial z^{K}}$$

Subsequently, the gradient of the loss function can be transformed as follows:

(13) $$\frac{\partial L(w,b,x,y)}{\partial w^{k-1}}=\frac{\partial L(w,b,x,y)}{\partial z^{k}}\frac{\partial z^{k}}{\partial w^{k-1}}=\delta^{k}(z^{k-1}{)}^{T}$$

(14) $$\frac{\partial L(w,b,x,y)}{\partial b^{k-1}}=\frac{\partial L(w,b,x,y)}{\partial z^{k}}\frac{\partial z^{k}}{\partial b^{k-1}}=\delta^{k}$$

Through mathematical derivation, the relationship between $\delta^{k}$ and $\delta^{k+1}$ can be obtained as follows:

(15) $$\delta^{k}=\delta^{k+1}\frac{\partial z^{k}}{\partial z^{k}}=(w^{k}{)}^{T}\delta^{k+1}⊙\sigma \prime (z^{k})$$

The changes in the weights and biases of the k-th layer due to gradient descent are as follows:

(16) $$w^{k}\leftarrow w^{k}-\alpha \sum_{j=1}^{n}{\delta_{j}}^{k}({z_{j}}^{k-1}{)}^{T}$$

(17) $$b^{k}\leftarrow b^{k}-\alpha \sum_{j=1}^{n}{\delta_{j}}^{k}$$

In this equation, α denotes the step size, and optimization functions that use gradient descent include SGD (Stochastic Gradient Descent), RMSprop (Root Mean Square Propagation), and Adam (Adaptive Moment Estimation) [28,29,30]. This study was not research on optimizing parameters; hence, the architecture was kept simple, and Adam was used as the optimization function. The architecture for predicting Loudness is listed in Table 4. 

The activation and optimization functions were defined as the ReLU function and Adam (learning rate: 0.001), respectively. Various methods have been developed for initial weights, such as Xavier and He initialization, to minimize the occurrence of convergence problems and local minimum issues [31,32]. However, since the aim of this study was not to optimize machine learning models, initialization issues were not addressed, and hyperparameters were not optimized. The dataset is divided into three parts: training, validation, and the test dataset. The dataset split ratio and the number of data points used for training are listed in Table 5. Since the order of data can also have a significant impact on the accuracy of training, data were shuffled to prevent overfitting due to sequential data. The input data were standardized to eliminate errors because of varying data sizes. The training was performed 1000 times, and early stopping was applied, which stops the training if there is no improvement in error after training 20 times. 

## 3. Results

### 3.1. Multiple Linear and Nonlinear Regression Results

As explained in Section 2.3, 5-fold cross-validation was performed, and the model with the lowest mean absolute error (MAE) was selected as the regression model. The independent variables were determined using correlation analysis, and sound pressure level and fluctuation strength were selected as the independent variables. The result of the final selected regression equation is as follows:

(18) $$y=-36.62+0.75x_{1}-15.54x_{2}\;,\;\;\;\;\;\left\{\begin{array}{c} y=Loudness\\ x_{1}=Sound\;\;Pressure\;\;L\;evel\\ x_{2}=\;Fluctuation\;\;Strength \end{array}\right.$$

In this equation, the coefficients of the final selected linear regression equation are shown in Equation (18), where $x_{1}$ denotes sound pressure level and $x_{2}$ denotes fluctuation strength. The significance level of the F-statistic was ≤0.05, and the coefficient of determination ($R^{2}$) was 0.81, confirming the significance of the regression equation. The significance levels of the *p*-value test for the two variables were ≤0.05, verifying the significance of the regression coefficients. However, the mean relative error of the regression Equation (18) was 38.59%. Therefore, the results suggest that the linear regression model is incapable of predicting results, including noise. 

**Figure 7:**
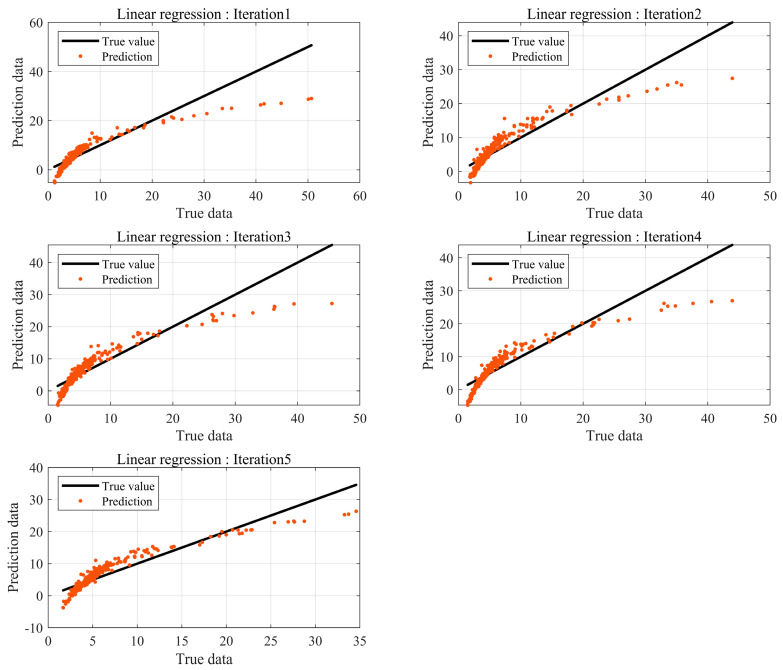
Prediction error by linear regression.

As illustrated in Figure 7, the results of the multiple linear model showed a relatively large error. Thus, the results suggest that the multiple linear model is not suitable for Loudness estimation, and the regression equation was derived using a nonlinear model as follows: Equation (18) shows the regression coefficients of the nonlinear model, where $x_{1}$ denotes sound pressure level and $x_{2}$ denotes fluctuation strength.

(19) $$y=e^{-18.31}\times {x_{1}}^{4.82}{x_{2}}^{-0.12},\left\{\begin{array}{c} y\;\;=\;Loudness\\ \;x_{1}\;=\;Sound\;Pressure\;L\;evel\\ x_{2}\;=\;Fluctuation\;Strength \end{array}\right.\;$$

**Figure 8:**
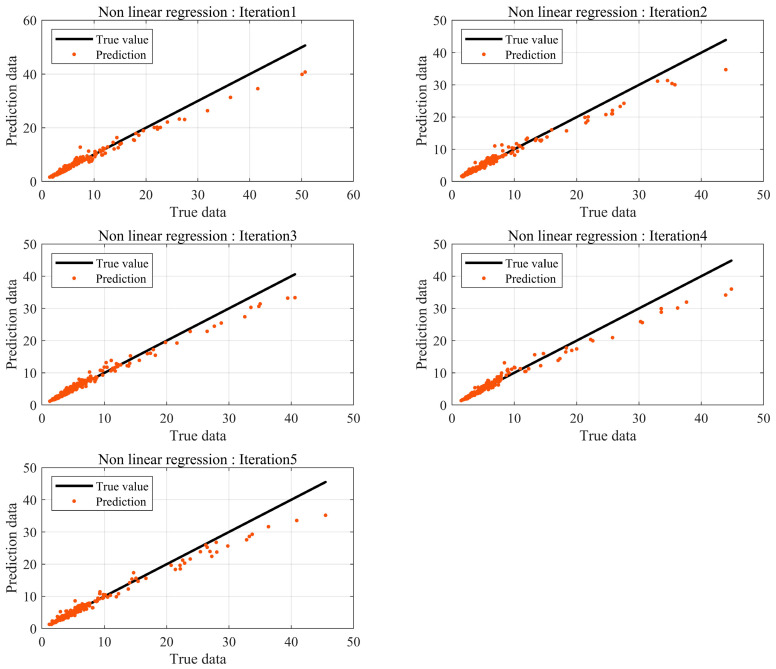
Prediction error by nonlinear regression.

The significance level of the F-statistic was ≤0.05. Additionally, $R^{2}$ was 0.97, confirming the significance of the regression equation. The significance levels of the *p*-value test for the two variables were both ≤0.05, confirming the significance of the regression coefficients. The mean relative error of Equation (19) on the test dataset was 8.9%. As shown in Figure 8, the results based on the nonlinear regression equation showed relatively higher prediction accuracy compared to the predictions estimated using the linear regression equation. However, since the quantitative BSR evaluation method determines Fail or Pass within a one sone range, high accuracy is required. Therefore, the prediction model needs to generate more precise results. Consequently, a prediction model using a DNN was constructed.

### 3.2. Loudness Prediction Results Based on Machine Learning 

**Figure 9:**
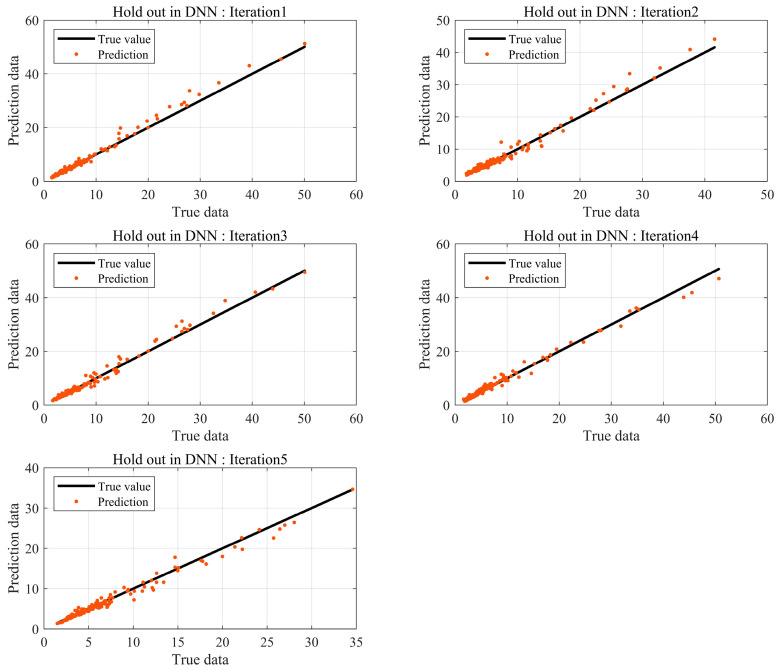
Prediction error by DNN using hold out.

The Loudness prediction was performed using machine learning based on the same data used in K-fold cross-validation. The traditional data partitioning method is a hold-out, where data are randomly categorized into training and testing datasets, using the training data to train the model. If there is sufficient data, the hold-out method can be used to construct a sophisticated model. Otherwise, a model created may not fully represent the entire dataset. Figure 9 illustrates the test results of the DNN model using hold-out. Relatively large errors occur in results with high Loudness levels. BSR from automotive seats primarily occurs at or below 10 sones; hence, results at or above 25 sones can be considered outliers from a statistical perspective. The data distribution shows prediction errors tend to be closer to the actual values compared to regression-based prediction errors. 

**Figure 10:**
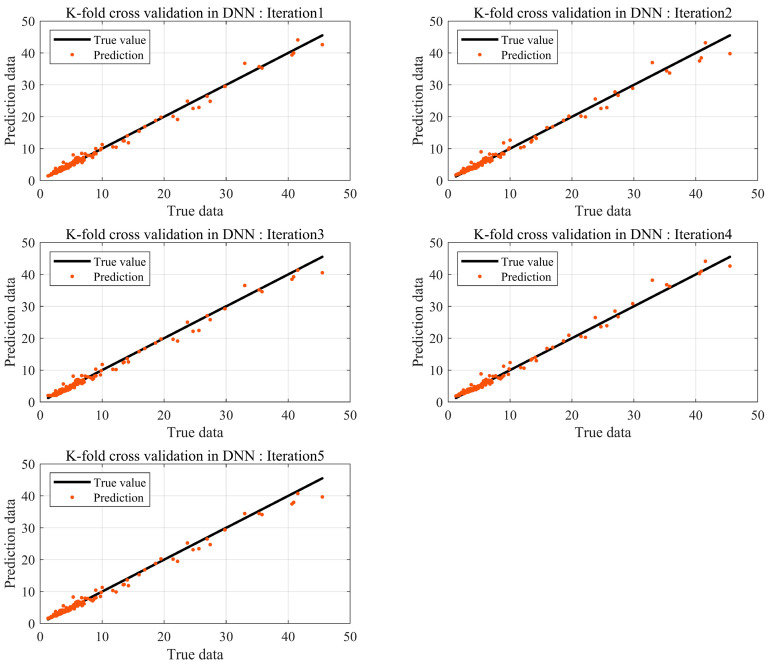
Prediction error by DNN using K-fold cross-validation.

In machine learning, the number of data has a significant impact on the performance of the model, provided the model avoids overfitting and underfitting problems. BSR experiments are conducted at the final phase of development, and not all products are tested. Consequently, it is challenging to obtain substantial data through experiments. Thus, prediction models are needed to achieve robust interpretation accuracy with a small amount of data. Data augmentation based on data transformations is a common method for acquiring data. However, they occur in data augmentation and must be used appropriately according to the purpose and context. Therefore, in this study, K-fold cross-validation was utilized, which can create stable models using a small amount of data without distorting the data. The results are shown in Figure 10. 

**Figure 11:**
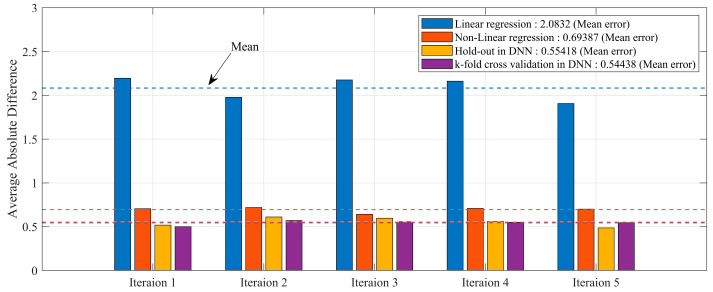
Comparing the performance of each model.

Compared to methods that incorporated the linear and nonlinear regression equations along with the hold-out method, the DNN that utilized K-fold cross-validation predicted results that were closer to the actual values. The absolute errors of all results are shown in Figure 11. 

**Figure 12:**
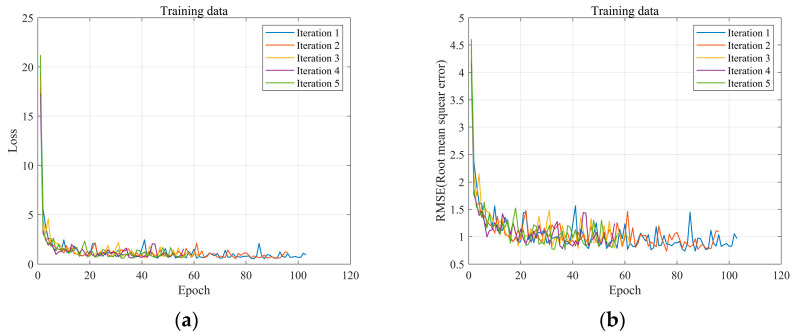
Results of the experiment: (**a**) loss: (**b**) root mean square.

The mean absolute error was relatively the lowest for the K-fold cross-validation in DNN, with a value of 0.54, while it was highest for linear regression, with a value of 2.08. In this study, the hold-out method in DNN did not establish a validation dataset; hence, a relatively large dataset was used. This indicates that the validation data cannot be used for training. With limited data, overfitting and underfitting are common problems in the model, necessitating data augmentation. Therefore, the proposed model applied K-fold cross-validation to the DNN model to solve the issue with the number of data points and predict Loudness by incorporating all data trends. Although the absolute errors for the hold-out method and K-fold cross-validation are similar, the deviation is likely to be large if the data are small. The proposed model aimed to solve the issue with the number of data points and predict Loudness by incorporating all data trends based on the K-fold cross-validation in DNN. The value of K was defined as 5 in the model used. Figure 12 illustrates the training process. 

As illustrated in Figure 12a,b, the training progress result was similar to that of most machine learning models. The loss is initially large but quickly converges as the training proceeds. This indicates that although BSR is a phenomenon with characteristics that are difficult to physically investigate and elucidate, it can be predicted relatively easily using machine learning. 

## 4. Conclusions

In this study, we aimed to propose a deep learning method for predicting Loudness N10, a quantitative metric for BSR. This metric requires difficult conditions and is complex to calculate based on the physical quantities related to the acoustics and sound quality of automotive seats for sound sources containing noise. Among various physical quantities, sound pressure level and fluctuation strength were derived as significant factors based on the analysis of variance results. In addition, the traditional K-fold cross-validation method was utilized to derive linear and nonlinear regression equations. However, the prediction results showed relatively large errors, with values of 2.08 and 0.69. This outcome indicates that BSR cannot be predicted using regression equations. 

Conversely, predictions using DNN in hold-out estimated Loudness accurately, with a value of 0.55. We obtained numerous datasets from other studies. However, it is nearly impossible to acquire a large amount of data and various types of datasets from experiments. The K-fold cross-validation method can achieve maximum efficiency within a limited dataset for development purposes and from a methodology perspective. Therefore, we proposed the method of applying K-fold cross-validation to a DNN as a method of predicting Loudness. Consequently, we attained the best-performing prediction model within an error range of 0.54. Since we could not acquire extensive BSR noise datasets in a limited environment, we utilized the proposed DNN method to verify that the proposed model has relatively superior performance. It is predicted that the quantitative test index for BSR can be estimated using a few sound-quality physical quantities, even when noise is included. Therefore, the results of this study suggest that it is feasible to estimate the results of complex noise and vibration experiments, including BSR experiments with limited datasets. This demonstrates the significance of applying machine learning-based prediction methods to various engineering experiments that involve nonlinearity.

In future research, we aim to establish a methodology that utilizes several physical quantities to apply machine learning so that the BSR characteristics of the seat can be estimated from all positions in actual tests.

## Figures and Tables

**Table 1:** Normalized value of #D1.

Metrics	P1	P2	P3	P4	P5	P6	P7	P8	P9
M1	0.28	1.00	0.42	0.37	0.66	0.24	0.54	0.00	0.22
M2	0.48	1.00	0.26	0.47	0.77	0.35	0.60	0.00	0.23
M3	0.47	1.00	0.44	0.51	0.76	0.38	0.68	0.00	0.21
M4	0.66	1.00	0.49	0.66	0.88	0.54	0.82	0.00	0.00
M5	0.45	1.00	0.44	0.60	0.93	0.42	0.59	0.00	0.15
M6	0.36	1.00	0.11	0.11	0.21	0.01	0.19	0.00	0.38
M7	0.14	0.00	0.39	0.22	0.46	0.17	0.04	1.00	0.38
M8	0.57	1.00	0.27	0.35	0.38	0.18	0.00	0.25	0.65
M9	0.65	0.00	0.77	0.74	0.50	0.85	0.54	1.00	0.84
M10	0.77	0.00	0.88	0.89	0.78	0.95	0.73	1.00	0.81

**Table 2:** Analysis of variance (ANOVA) of #D1.

	M1	M2	M3	M4	M5	M6	M7	M8	M9	M10
M1	0.08									
M2	0.07	0.08								
M3	0.07	0.08	0.08							
M4	0.08	0.09	0.09	0.11						
M5	0.08	0.08	0.08	0.10	0.09					
M6	0.06	0.06	0.05	0.04	0.05	0.08				
M7	−0.05	−0.05	−0.06	−0.07	−0.05	−0.04	0.08			
M8	0.04	0.04	0.03	0.01	0.03	0.07	−0.02	0.08		
M9	−0.07	−0.07	−0.07	−0.07	−0.07	−0.07	0.05	−0.05	0.08	
M10	−0.07	−0.06	−0.06	−0.06	−0.06	−0.08	0.04	−0.06	0.07	0.08

**Table 3:** Correlation analysis of # s1.

	M1	M2	M3	M4	M5	M6	M7	M8	M9	M10
M1	1.00									
M2	0.95	1.00								
M3	0.95	0.98	1.00							
M4	0.83	0.91	0.94	1.00						
M5	0.90	0.96	0.97	0.94	1.00					
M6	0.83	0.71	0.65	0.43	0.55	1.00				
M7	−0.66	−0.68	−0.71	−0.70	−0.61	−0.49	1.00			
M8	0.56	0.45	0.36	0.14	0.33	0.88	−0.24	1.00		
M9	−0.99	−0.95	−0.93	−0.80	−0.87	−0.86	0.61	−0.60	1.00	
M10	−0.93	−0.80	−0.77	−0.59	−0.68	−0.96	0.53	−0.75	0.94	1.00

**Table 4:** Architecture of DNN.

Layer	Output Shape	Param #
Input Layer	(None, 2)	0
Dense 1	(None, 512)	1536
Dense 2	(None, 256)	131,328
Dense 3	(None, 128)	32,896
Dense 4	(None, 64)	8256
Dense 5	(None, 32)	2080
Output	(None, 1)	33
Total params:	176,129	
Trainable params:	176,129	
Non-trainable params:	0	

**Table 5:** Dataset split ratio.

Dataset	Percentage	Number of Samples
Training	64%	783
Validation	16%	196
Testing	20%	245

## Data Availability

Data are contained within the article.
